# Supplementary material for: Layer-specific optogenetic activation of pyramidal neurons causes beta–gamma entrainment of neonatal networks
Source: Nat Commun. 2017 Feb 20;8:14563. doi: 10.1038/ncomms14563 (PMC5321724; doi:10.1038/ncomms14563)
Supplement: Supplementary Information — Supplementary Figures [file ncomms14563-s1.pdf]

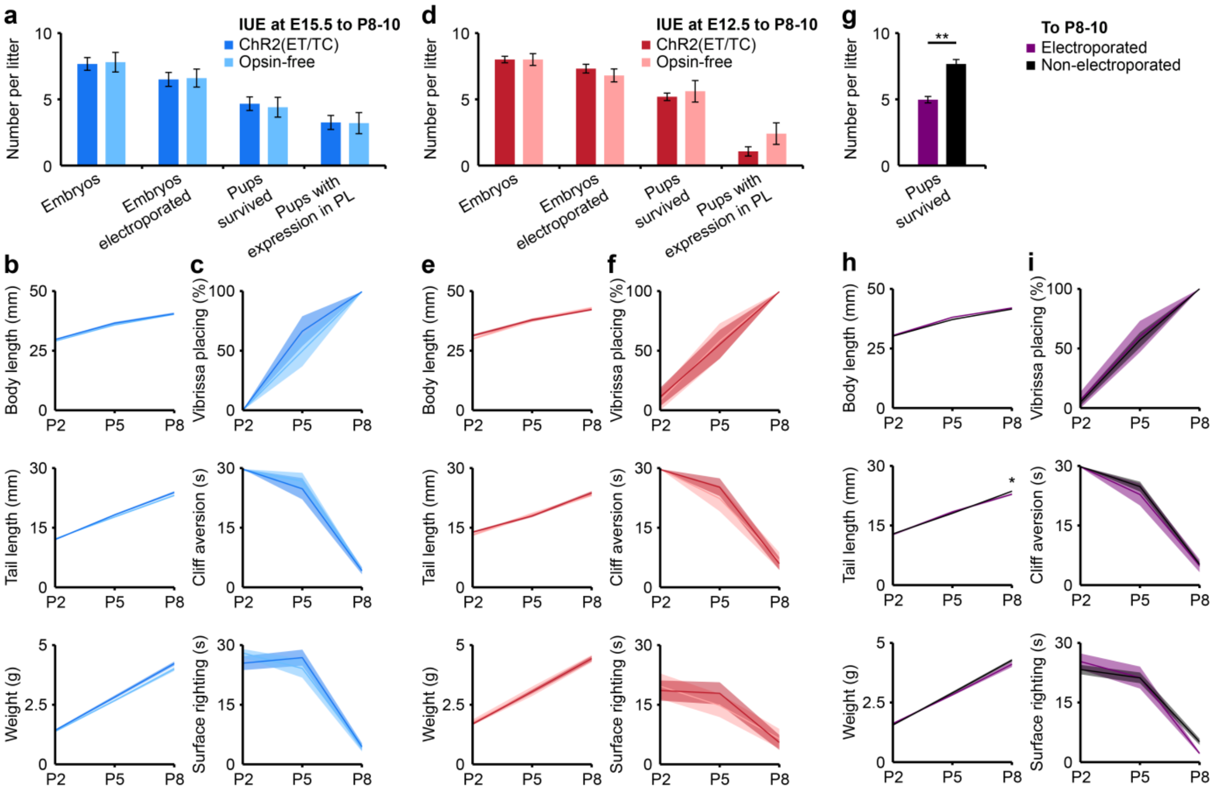

Supplementary Figure 1 (related to Figure 1): Somatic and reflex development of mouse pups transfected by *in utero* electroporation. **(a)** Bar diagram displaying the mean number of embryos, electroporated embryos, surviving pups, and positively transfected pups when ChR2(ET/TC)-containing (dark blue, n=12 litter) and opsin-free constructs (light blue, n=5 litter) were tranfected by IUE at E15.5. **(b)** Line plots displaying the developmental profile of somatic growth [body length (top), tail length (middle), weight (bottom)] of P2-8 pups expressing ChR2(ET/TC) (dark blue, n=17 pups) or opsin-free constructs (light blue, n=16 pups) in layer II/III pyramidal neurons. **(c)** Line plots displaying the development profile of reflexes [vibrissa placing (top), cliff aversion (middle) and surface righting reflexes (bottom)] of P2-8 pups expressing ChR2(ET/TC) (n=17, dark blue) or opsin-free constructs (n=16, light blue) in layer II/III pyramidal neurons. **(d)-(f)** Same as (a)-(c) for pups expressing ChR2(ET/TC)-containing (dark red, n=16 litter, n=18 pups) or opsin-free constructs (light red, n=5 litter, n=12 pups) in layer V/VI pyramidal neurons. **(g)** Bar diagram displaying the survival rate of electroporated (violet) and non-electroporated litters (black). **(h)-(i)** Same as (b)-(c) for electroporated (violet, n=63 pups) and non-electroporated pups (black, n=15 pups). Data are displayed as mean ± SEM. *p < 0.05, **p < 0.01, and ***p < 0.001, two-sided t-tests.

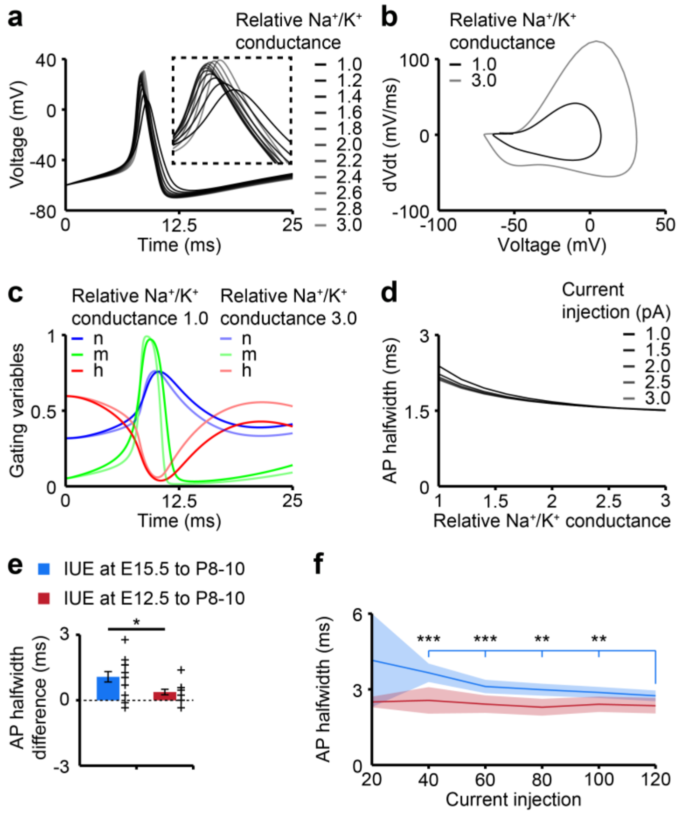

Supplementary Figure 2 (related to Figure 2,3): Control of AP shape by Na$^+$/K$^+$ conductance and input strength. **(a)** Dependence of AP time course on augmenting Na$^+$/K$^+$ conductance as modeled by the Hodgkin-Huxley conductance based model. **(b)** Phase plots of modeled APs for low (Na$^+$: 0.6 mS/cm^2; K$^+$: 0.18 mS/cm^2) and high (Na$^+$: 1.8 mS/cm^2; K$^+$: 0.54 mS/cm^2) Na$^+$/K$^+$ conductance. **(c)** Time course of the gating variables m, n and h of the differential equations of the Hodgkin-Huxley model. **(d)** Line plots displaying the relationship between AP half-width and current input for different Na$^+$/K$^+$ conductances. **(e)** Bar plot displaying the difference between half-width of APs triggered by current and by light for layer II/III (blue, n=13 neurons) and layer V/VI (red, n=12 neurons) pyramidal neurons. **(f)** Line plots displaying the relationship between APs half-width and injected current for transfected layer II/III (blue, n=13) and V/VI (red, n=12) pyramidal neurons. Data are presented as mean ± SEM. *$p < 0.05$, **$p < 0.01$, and ***$p < 0.001$, two-sided t-test.

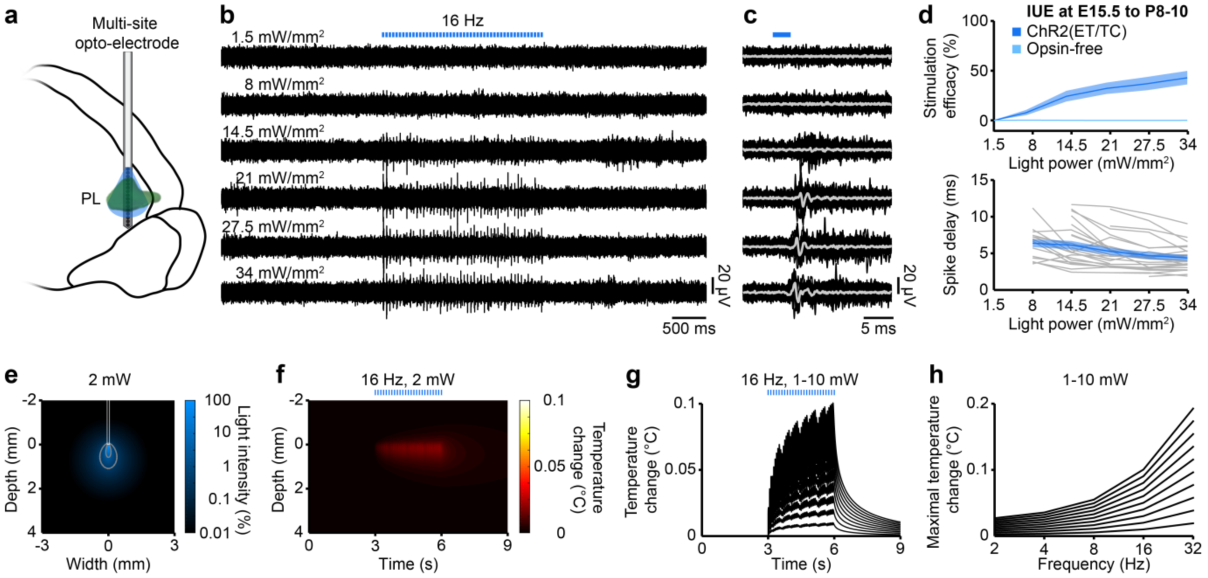

Supplementary Figure 3 (related to Figure 4): Assessment of spatiotemporal dynamics of light and heat propagation for *in vivo* optogenetics in the neonatal brain. **(a)** Schematic representation of combined light stimulation and extracellular recordings in the neonatal PL *in vivo*. **(b)** Representative MUA (band-pass filter: 500 to 5000 Hz) at one recording site in response to trains of light stimuli (473 nm, 3 ms-long, 16 Hz, total duration 3 s) at six levels of light intensity. **(c)** Superimposed single (black) and mean (gray) traces of representative MUA evoked by 30 individual light pulses (473 nm, 3 ms) at six levels of light intensity aligned at stimulus onset. **(d)** Mean stimulation efficacy (top) and spike delay (bottom) in response to trains of light stimuli (473 nm, 3 ms, 16 Hz) at six levels of light intensity when averaged for ChR2(ET/TC)-transfected (n=29 recording sites from 10 pups) and opsin-free (n=11 recording sites from 11 pups) pups. Gray lines correspond to individual recording channels. **(e)** Propagation of light intensity in the brain as predicted by Monte Carlo simulations for the used optical fiber (diameter 105 µm, numerical aperture 0.22, light parameters: 473 nm, 2 mW). Gray lines correspond to the iso-contour lines for 1 and 10 mW/mm². **(f)** Color-coded map of predicted heat-changes over time and tissue depth for trains of light pulses (473 nm, 3 ms) at 16 Hz for 3 s and 2 mW. **(g)** Line plot displaying the temperature change below the fiber tip over time for light pulses (473 nm, 3 ms, 16 Hz) with increasing power (1 to 10 mW). **(h)** Line plot displaying the relationship between maximal temperature change and frequency of light stimulation for increasing light power (1 to 10 mW). Data were obtained from pups expressing ChR2(ET/TC) in layer II/III pyramidal neurons and are displayed as mean ± SEM.

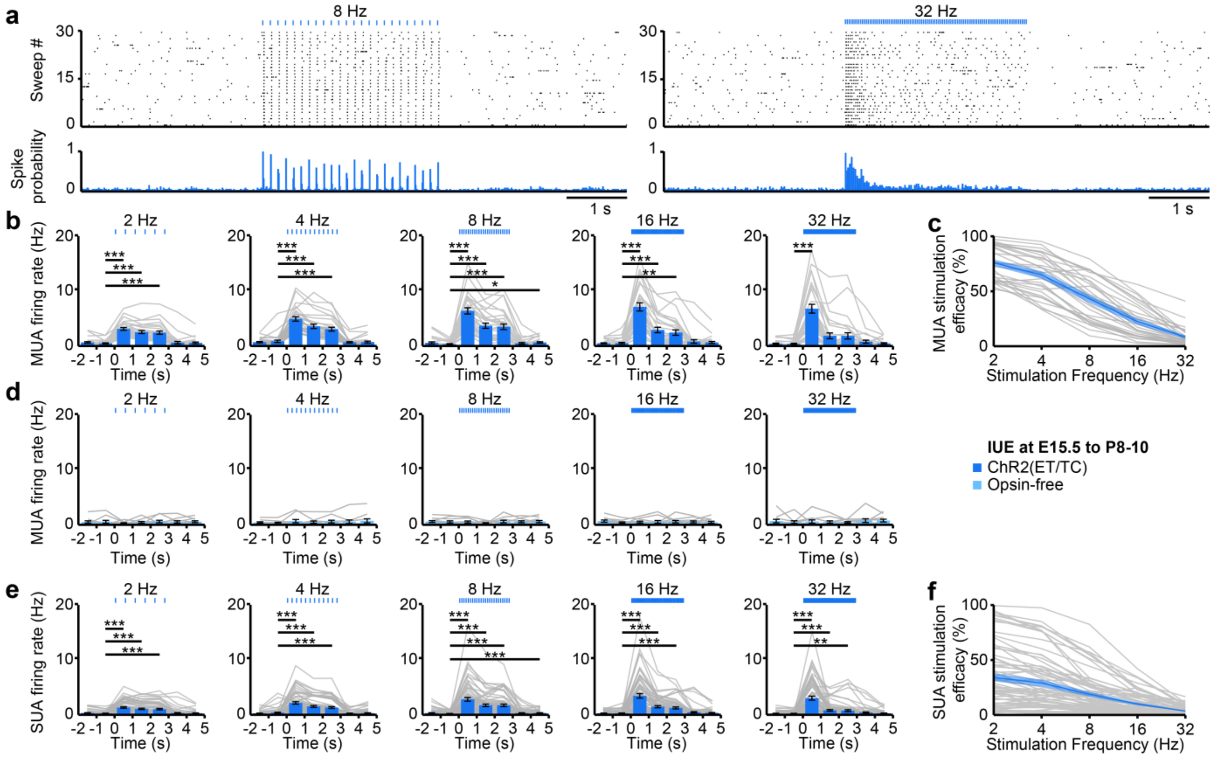

Supplementary Figure 4 (related to Figure 4): Optogenetic activation of layer II/III pyramidal neurons in response to pulse train stimulation *in vivo*. **(a)** Representative raster plots and corresponding spike probability histograms displaying the firing of ChR2(ET/TC) transfected layer II/III pyramidal neurons in response to 30 sweeps of illumination (473 nm, 3 ms) at 8 Hz (left) and 32 Hz (right). **(b)** Bar diagrams displaying the mean MUA firing rate of ChR2(ET/TC)-transfected neurons from P8-10 mice before, during and after trains of pulsed light stimulation (473 nm, 3 ms) at 2, 4, 8, 16, and 32 Hz (n=29 recording sites from 10 mice). **(c)** Mean (dark blue) and individual (gray) stimulation efficacy of MUA in response to pulsed light stimulation (473 nm, 3 ms) at the six different frequencies (n=29 recording sites from 10 pups). **(d)** Same as (b) averaged for P8-10 mice expressing opsin-free constructs in layer II/III pyramidal neurons (light blue, n=11 recording sites from 11 pups). **(e)** Bar diagrams displaying the mean SUA firing rate of ChR2(ET/TC)-transfected neurons from P8-10 mice before, during and after trains of pulsed light stimulation (473 nm, 3 ms) at 2, 4, 8, 16, and 32 Hz (n=69 recording sites from 10 pups). **(f)** Mean (dark blue) and individual (gray) stimulation efficacy of SUA in response to pulsed light stimulation (473 nm, 3 ms) at the six different frequencies (n=69 recording sites from 10 pups). Data are presented as mean ± SEM. *p < 0.05, **p < 0.01, and ***p < 0.001, one-way repeated measures ANOVA with Bonferoni corrected post hoc analysis.

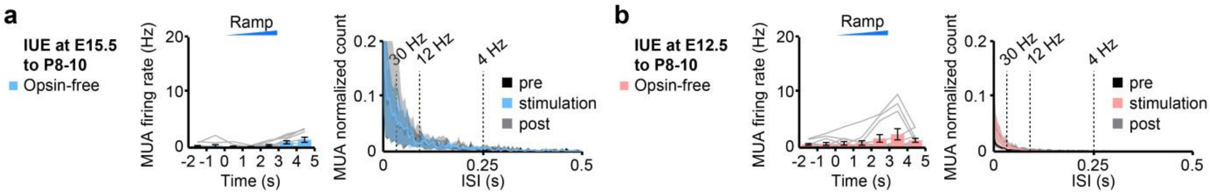

Supplementary Figure 5 (related to Figure 4): *In vivo* spiking of layer II/III and layer V/VI pyramidal neurons transfected by IUE with opsin-free constructs. **(a)** Left, bar diagram displaying the mean MUA frequency in response to ramp illumination averaged for mice expressing opsin-free constructs in layer II/III pyramidal neurons (light blue). Right, occurrence rate of inter-spike intervals averaged for 3 s before (pre, black), 3 s during (stimulation, blue), and 3 s after ramp stimulation (post, gray) (n=11 recording sites of 11 pups). **(b)** Same as (a) for mice expressing opsin-free constructs in layer V/VI (light red, n=12 recording sites of 12 pups). Gray lines correspond to individual firing rates. Data are presented as mean ± SEM. *p < 0.05, **p < 0.01, and ***p < 0.001, one-way repeated measures ANOVA with Bonferoni corrected post hoc analysis.

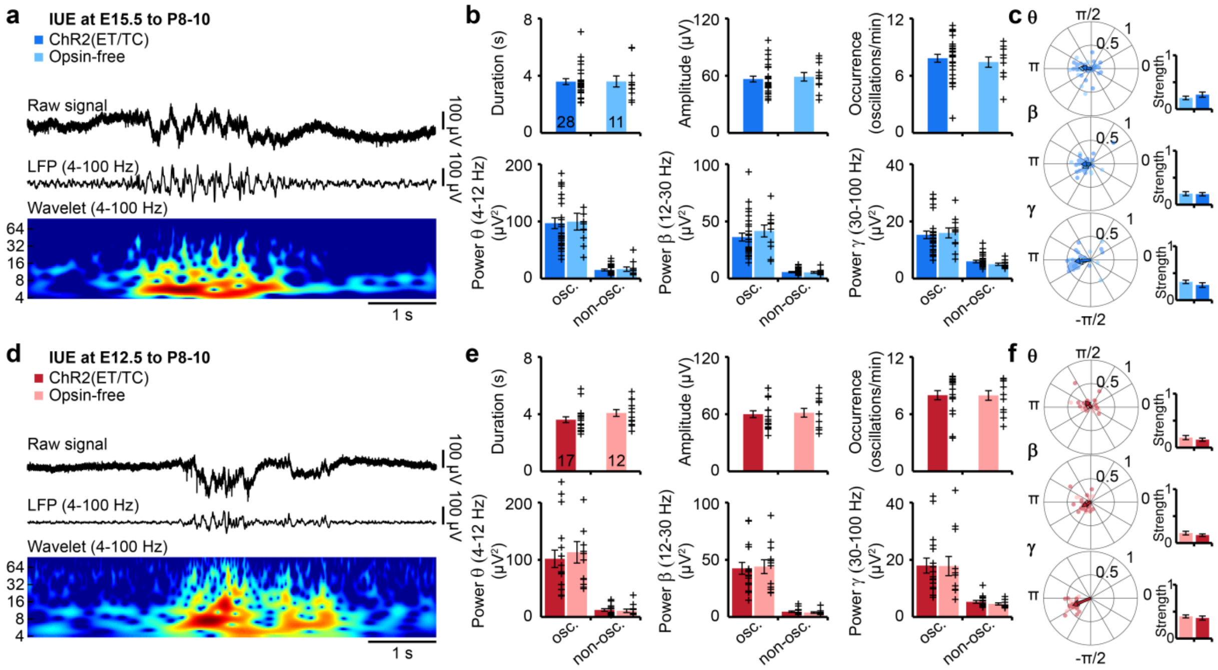

Supplementary Figure 6 (related to Figure 5,6): Spontaneous oscillatory activity of ChR2(ET/TC) and opsin-free transfected mice. **(a)** Characteristic burst of spontaneous discontinuous oscillatory activity from a P10 mouse after transfection of layer II/III pyramidal neurons with ChR2(ET/TC) by IUE. The nested gamma spindle burst is displayed before (top) and after band pass filtering (4-100 Hz) (middle) together with the corresponding color-coded wavelet spectrum at identical time scale. **(b)** Bar diagrams displaying the properties of spontaneous oscillatory activity averaged for layer II/III ChR2(ET/TC)-transfected (dark blue, n=28 pups) and opsin-free mice (light blue, n=11 pups). Note the different scaling for theta (θ, 4-12 Hz), beta (β, 12-30 Hz), and gamma (ɣ, 30-100 Hz) power. Individual values corresponding to pups with light-induced network oscillations are displayed as black crosses. **(c)** Polar plots displaying the phase locking of spontaneous MUA to oscillatory activity (top: θ; middle: β; bottom: ɣ frequency band) from layer II/III ChR2(ET/TC)-transfected (dark blue, n=28 pups) and opsin-free mice (light blue, n=11 pups). The values from individual pups with light-induced network oscillations are shown as dark and light blue dots, whereas the arrows correspond to the mean resulting group vectors. Bar diagrams display the mean locking strength. **(d)-(f)** Same as (a)-(c) for layer V/VI ChR2(ET/TC)-transfected (dark red, n=17 pups) and opsin-free mice (light red, n=12 pups). For (b) and (e) osc = oscillations, non-osc = time windows without oscillatory activity. Data are presented as mean ± SEM. *p < 0.05, **p < 0.01, and ***p < 0.001, two-sided t-tests and circular statistics toolbox.

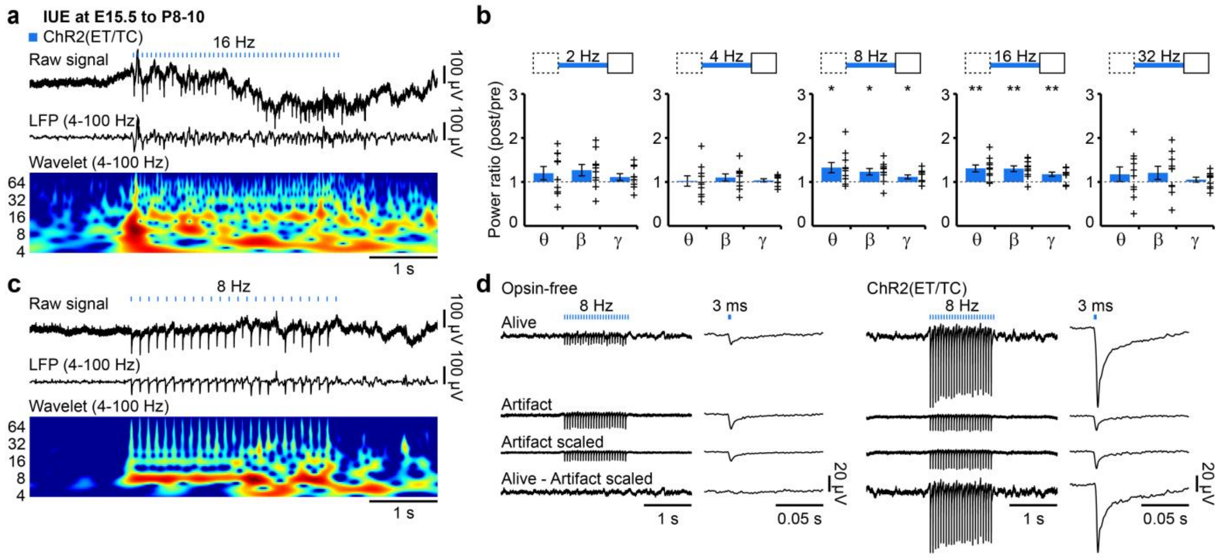

Supplementary Figure 7 (related to Figure 5): Methodological approach for elimination of photoelectric artifacts. **(a)** Characteristic LFP in the PL of a layer II/III expressing P10 mouse in response to a train of light stimuli at 16 Hz (473 nm, 3 ms). The LFP is displayed before (top) and after band-pass filtering (4-100 Hz) (middle) together with the corresponding color-coded wavelet spectrum at identical time scale. **(b)** Bar diagrams displaying the LFP power ratio before (pre, 1.5 s) and after (post, 1.5 s) trains of light stimuli (473 nm, 3 ms-long, total duration 3 s) at 2, 4, 8, 16 or 32 Hz for the main frequency bands of neonatal oscillatory activity (θ, β, ɣ) (n=10 pups). Individual values corresponding to pups with light-induced network oscillations are displayed as black crosses. **(c)** Characteristic LFP in the PL of a P10 mouse transfected by IUE at E15.5 with ChR2(ET/TC) in response to a train of light stimuli (473 nm, 3 ms) at 8 Hz. The LFP is displayed before (top) and after band-pass filtering (4-100 Hz) (middle) together with the corresponding color-coded wavelet spectrum at identical time scale. **(d)** Representative band-pass filtered (1-400 Hz) averaged LFP recorded in response to 30 trains of light stimuli (473 nm, 3 ms, 8 Hz) or single stimuli before (Alive, top) and after (Artifact, Artifact scaled, middle) a lethal injection of ketamine-xylazine from a mouse transfected by IUE with the opsin-free construct (left) and from a mouse transfected with ChR2(ET/TC) (right). The LFP trace after removal of photoelectric artifacts was obtained by subtraction of the scaled artifact (Alive-Artifact scaled, bottom). Note the presence of prominent current sinks generated by the simultaneous opening of light-activated channels only in the ChR2(ET/TC)-expressing animal. Data are presented as mean ± SEM. $*p < 0.05$, $**p < 0.01$, and $***p < 0.001$, two-sided t-tests.

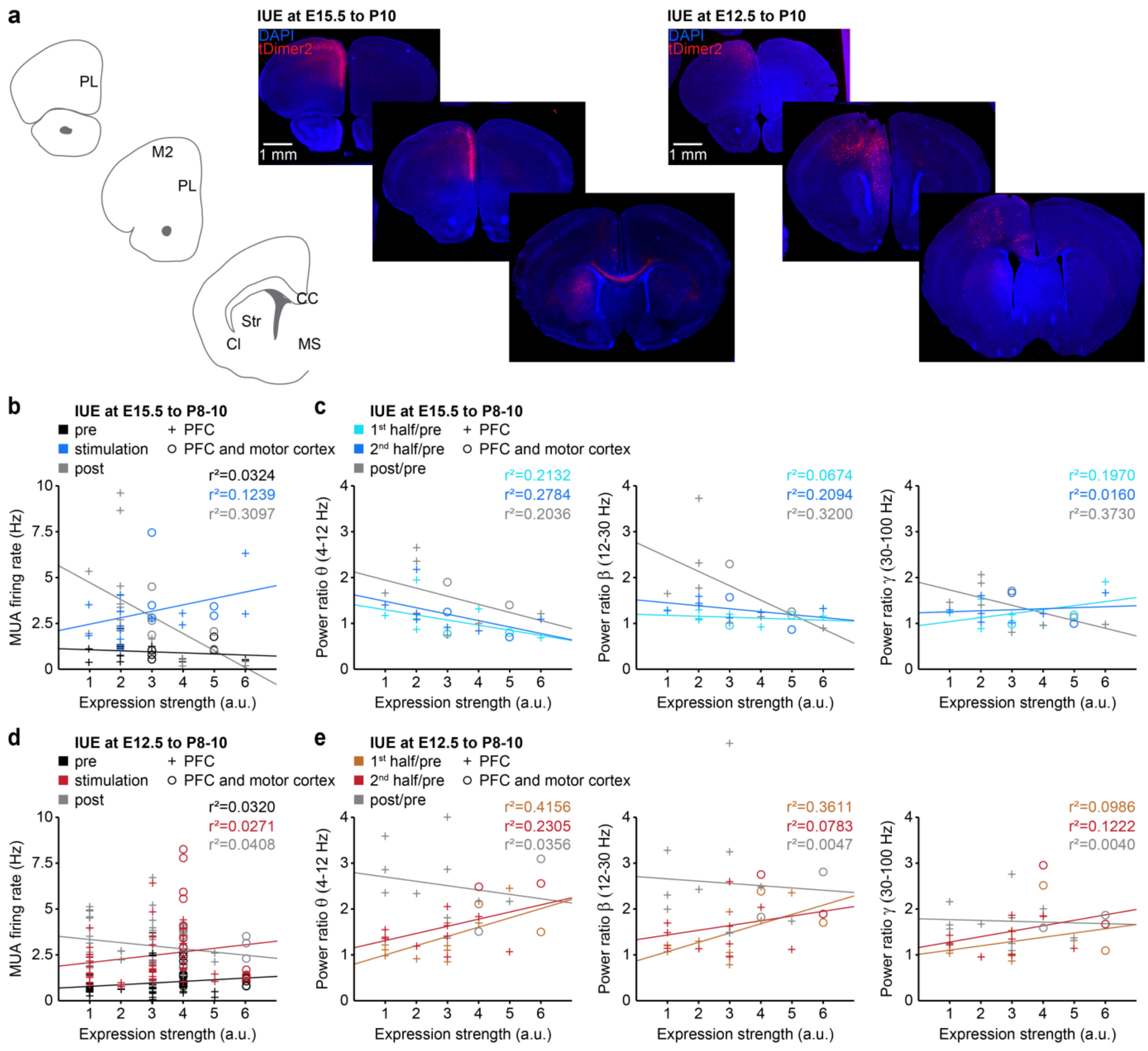

Supplementary Figure 8 (related to Figure 5,6): Impact of light stimulation on neuronal and network activity in relationship to expression strength of ChR2(ET/TC). **(a)** tDimer2-expressing cells (red) in DAPI-stained (blue) coronal sections (50 µm-thick) of two P10 mice after E15.5 (left) and E12.5 (right) IUE, respectively. Note the presence of transfected neurons in PFC and motor cortex as well as the presence of positive fibers (e.g. in striatum). **(b)** Scatter plot with linear regression displaying the MUA firing rate before (pre 3 s, black), during (stimulation 3 s, blue) and after (post 3 s, gray) ramp stimulation in relation to the expression strength for layer II/III expressing P8-10 mice. Values of individual recording sites are displayed as crosses for pups expressing the construct only in the PFC (n=16 recording sites, n=7 pups) and as circles for pups with expression both in the PFC and motor cortex (n=5 recording sites, n=2 pups). **(c)** Scatter plots with linear regressions displaying baseline-normalized LFP power during first half (1st half), second half (2nd half) and after (post) ramp stimulus in relation to the expression strength in layer II/III of P8-10 mice Theta (θ), beta (β) and gamma (γ) frequency bands were considered for analysis. Values corresponding to pups with PFC-confined expression (n=7 pups) are displayed as crosses, whereas values from pups with expression both in PFC and motor cortex (n=2 pups) are shown as circles. **(d)-(e)** Same as (b)-(c) for layer V/VI neurons (PFC n=49 recording sites, n=10 pups; PFC and motor cortex n=12 recording sites, n=2 pups). Data are presented as mean for each recording site.

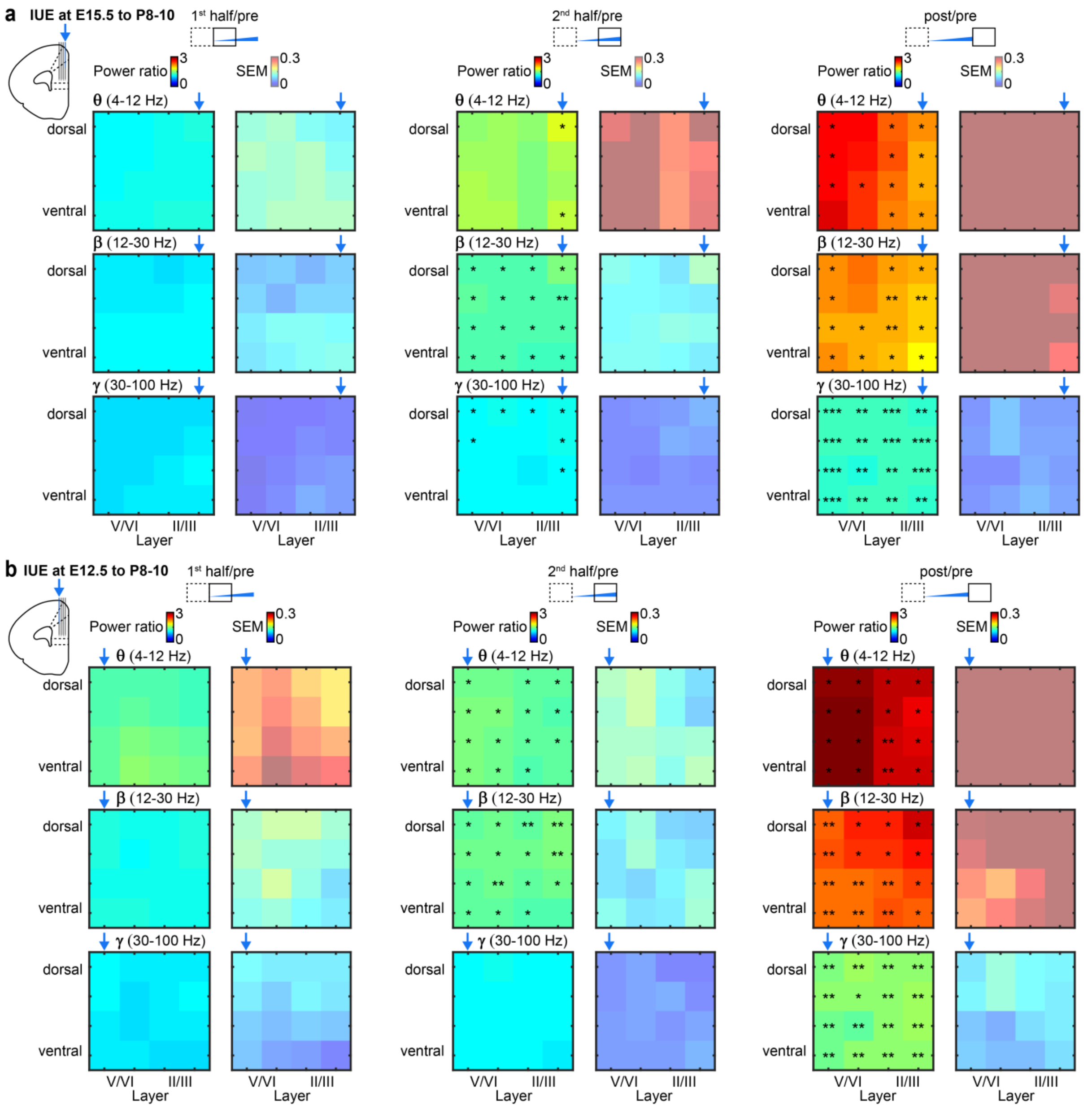

Supplementary Figure 9 (related to Figure 7): Frequency-dependent activation of neonatal prelimbic cortex in response to layer-specific optogenetic activation *in vivo*. **(a)** Color-coded images displaying the baseline normalized (pre, 1.5 s) power (mean and SEM) in response to light stimulation of layer II/III pyramidal neurons for recording sites spanning the PL depth during the first half (1st half, 1.5 s), the second half (2nd half, 1.5 s), and after (post, 1.5 s) ramp stimulus. The power was calculated for theta (θ, 4-12 Hz, top), beta (β, 12-30 Hz, middle) and gamma (ɣ, 30-100 Hz, bottom) frequency bands and values were averaged (n=11 pups). **(b)** Same as (a) for layer V/VI expressing P8-10 mice (n=6). Blue arrows indicate the position of the light fiber. *$p < 0.05$, **$p < 0.01$, and ***$p < 0.001$, two-sided t-tests.
